# Club Cell Secretory Protein in Lung Disease: Emerging Concepts and Potential Therapeutics

**DOI:** 10.1146/annurev-med-042921-123443

**Published:** 2022-11-30

**Authors:** Tereza Martinu, Jamie L. Todd, Andrew E. Gelman, Stefano Guerra, Scott M. Palmer

**Affiliations:** 1 Department of Medicine, University of Toronto, Toronto, Ontario, Canada; email: tereza.martinu@uhn.ca; 2 Division of Respirology, Department of Medicine, University Health Network, Toronto, Ontario, Canada; 3 Toronto Lung Transplant Program, Ajmera Transplant Centre, University Health Network, Toronto, Ontario, Canada; 4 Division of Pulmonary, Allergy and Critical Care, Department of Medicine, Duke University Medical Center, Durham, North Carolina, USA; 5 Duke Clinical Research Institute, Durham, North Carolina, USA; 6 Department of Surgery, Washington University School of Medicine, St. Louis, Missouri, USA; 7 Asthma and Airway Disease Research Center, University of Arizona, Tucson, Arizona, USA; 8 Division of Pulmonary, Allergy, Critical Care and Sleep Medicine, Department of Medicine, University of Arizona College of Medicine, Tucson, Arizona, USA

**Keywords:** club cell secretory protein, CCSP, CC10, uteroglobin, secretoglobin 1A1, SCGB1A1, lung biology, biomarker

## Abstract

Club cell secretory protein (CCSP), also known as secretoglobin 1A1 (gene name *SCGB1A1*), is one of the most abundant proteins in the lung, primarily produced by club cells of the distal airway epithelium. At baseline, CCSP is found in large concentrations in lung fluid specimens and can also be detected in the blood and urine. Obstructive lung diseases are generally associated with reduced CCSP levels, thought to be due to decreased CCSP production or club cell depletion. Conversely, several restrictive lung diseases have been found to have increased CCSP levels both in the lung and in the circulation, likely related to club cell dysregulation as well as increased lung permeability. Recent studies demonstrate multiple mechanisms by which CCSP dampens acute and chronic lung inflammation. Given these anti-inflammatory effects, CCSP represents a novel potential therapeutic modality in lung disease.

## INTRODUCTION

Club cell secretory protein (CCSP) is a member of the secretoglobin family of proteins that are secreted primarily by club cells in the airway epithelium of terminal bronchioles. CCSP has also been referred to as CC16, based on its 16-kD molecular weight. The official protein name is secretoglobin 1A1 (gene *SCGB1A1*), as determined by a panel of experts in 2000 (1). Other names have included Clara cell secretory protein, CC10, CC17, uteroglobin, urine protein 1, protein 1, blastokinin, progesterone-binding protein, and polychlorinated biphenyl-binding protein. Emerging evidence described in this review, including preclinical, clinical, and genetic studies, demonstrates a critical role for CCSP as a regulator of lung inflammation. The goals of this review are to summarize current knowledge regarding CCSP, highlight its role in modulation of pulmonary physiology and biology, and explore its potential to serve as a diagnostic biomarker and even therapeutic modality in pulmonary medicine.

## CCSP BIOLOGY

### Cellular Sources of CCSP

CCSP, as the name implies, is most highly expressed in club cells, which are nonciliated epithelial cells lining the distal and proximal human lung epithelium. CCSP expression has also been detected in alveolar type 2 progenitor, goblet, nonmucous and nonciliated epithelial cells in human sino-nasal epithelium (2, 3). In mouse lungs, club cells are found only within bronchioles, which are the major source of pulmonary CCSP expression. Lower levels of mouse CCSP are also detected more distally within bronchioalveolar epithelial progenitor cells, which appear to be the functional equivalent of CCSP^+^ basal cells in human lungs (4). CCSP is produced constitutively under normal conditions and is one of the most abundant proteins in the lung.

**Figure 1:**
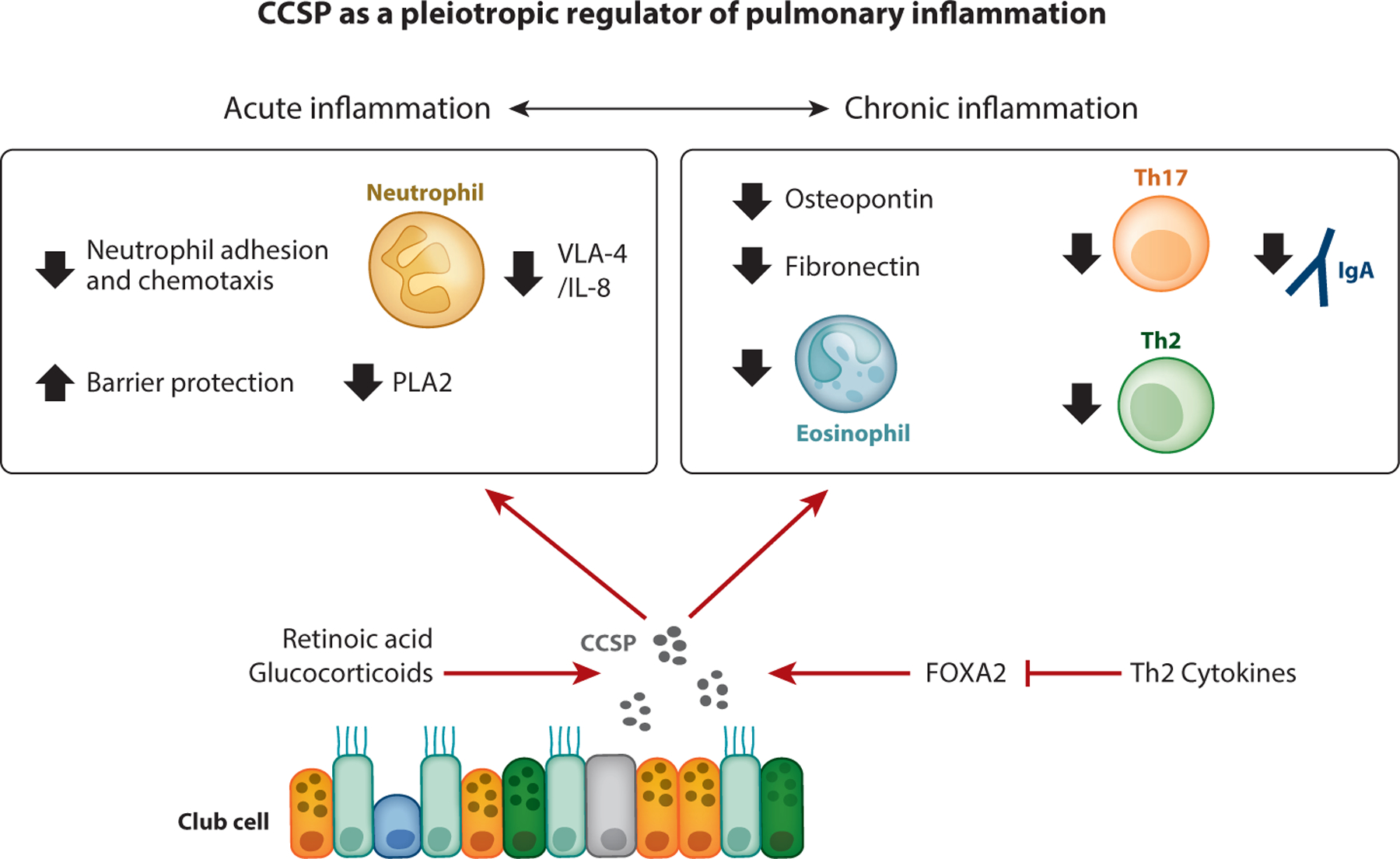
CCSP restrains pulmonary inflammation. CCSP expression is promoted by retinoic acid and glucocorticoids, as well as through the activity of the transcription factor FOXA2. CCSP inhibits acute lung injury by blunting neutrophil lung recruitment through antagonizing VLA-4-mediated endothelial adhesion or inhibiting IL-8-directed chemotaxis. CCSP promotes barrier integrity by reducing the activity of PLA2, an oxidized lipid generator that stimulates epithelial and endothelial permeability. CCSP also controls chronic pulmonary inflammation. CCSP inhibits eosinophilia and Th17 responses mediated by osteopontin expression and antigen presentation. Furthermore, CCSP inhibits Th2 cytokine expression, which in turn antagonizes FOXA2 expression. Finally, CCSP prevents humoral responses by inhibiting complex formation between IgA and fibronectin. Abbreviations: CCSP, club cell secretory protein; FOXA2, forkhead box A2; IgA, immunoglobulin A; IL-8, interleukin-8; PLA2, phospholipase A2; Th, helper T cell; VLA-4, very late antigen-4.

While the regulation of CCSP expression is incompletely understood, it is known that its production can be stimulated by glucocorticoids (5) and retinoic acid (6). Additionally, the transcription factor forkhead box A2 (FOXA2) promotes CCSP expression (7). FOXA2 expression is inhibited by type 2 helper T cell (Th2) cytokines, in line with reduced CCSP levels in asthma patients (8) (Figure 1).

CCSP mRNA has also been identified in human uterine, thymus, prostate, and pituitary gland tissues (9, 10), suggesting that extrapulmonary sources of CCSP could contribute to systemic levels. Human proteome maps have demonstrated CCSP expression primarily in lung epithelium (11). Interestingly, several studies in mice demonstrate CCSP-expressing bone marrow cells that promote lung repair (12). In humans undergoing allergen-specific immunotherapy, there are recent observations of sputum macrophages and lymphocytes upregulating CCSP and its mRNA (13).

Thus, while CCSP production has been mostly ascribed to club cells, non–club cell or extrapulmonary CCSP sources may play key roles in barrier organ homeostasis. Ongoing and future studies utilizing single-cell approaches will shed further light on CCSP expression patterns.

### Immunoregulation

Experimental and clinical studies demonstrate that CCSP is a negative regulator of acute and chronic pulmonary inflammation (Figure 1).

In the context of acute lung inflammation, CCSP has been shown to limit neutrophil recruitment. Adenoviral and respiratory syncytial viral infections exacerbate alveolitis and promote significantly more airway neutrophilia in CCSP-deficient mice than in wild-type mice (14, 15). Intratracheal administration of recombinant human CCSP (rhCCSP) to premature infants with respiratory distress syndrome was associated with decreases in airway neutrophilia and signs of edema (14). The precise mechanisms by which CCSP limits lung inflammation remain to be completely elucidated; however, emerging data have provided new insights. Antibody neutralization of CCSP in serum samples from trauma patients who later developed pneumonia was reported to increase responsiveness to neutrophil chemotaxis mediated by interleukin-8 (IL-8) (16). Further, chronic obstructive pulmonary disease (COPD) patient sputum was observed to have ratios of CCSP to IL-8 that negatively correlated with small airway neutrophilia (17). The latter study also demonstrated that rhCCSP directly bound to IL-8 to inhibit neutrophil chemotaxis and reversed neutrophil migratory behavior promoted by cigarette smoke–treated human airway epithelial cultures. A recent demonstration that the integrin very late antigen-4 (VLA-4) functions as a CCSP receptor indicates that CCSP may directly antagonize neutrophil adhesion to pulmonary endothelial cells (18). Another mode by which CCSP could inhibit acute lung injury is through antagonizing the effects of phospholipase A2 (PLA2) (19). PLA2 is best known for its role in the conversion of pulmonary surfactant into oxidized lipid mediators known to promote epithelial permeability and for its capacity to enhance neutrophil recruitment. Consistent with these observations, CCSP-deficient mice have elevated PLA2 activity and neutrophil recruitment following lipopolysaccharide- and ventilator-induced injury (20, 21). Additionally, lung-derived fluid samples from patients with acute respiratory distress syndrome (ARDS) contain less CCSP (22) and more PLA2 (23) than samples from non-ARDS mechanically ventilated patients.

CCSP also appears to play an immunoregulatory role in chronic airway inflammation. In an ovalbumin-induced mouse model of allergic rhinitis, eosinophilic inflammation and Th2 cytokine production were elevated in CCSP-deficient mice compared to wild-type mice (24). CCSP was found to regulate allergic rhinitis severity through inhibiting the expression and activity of osteopontin, an extracellular matrix protein that stimulates epithelial cell production of Th2 cytokines (24). Loss of CCSP expression has been particularly noted in nasal cells from allergic rhinitis patients and is consistent with the negative correlation observed between nasal CCSP levels and osteopontin expression (25). In separate reports using the ovalbumin-induced allergic rhinitis mouse model, CCSP was shown to inhibit eotaxin-mediated eosinophil recruitment through attenuating the expression of chitinase 3–like 1 (26) or through preventing dendritic cells from optimally promoting Th17 responses that augment eosinophilia (27). Finally, CCSP has been shown to inhibit the formation of a complex between the extracellular matrix protein fibronectin and immunoglobulin A (IgA) (28). Mice deficient in CCSP develop severe IgA nephropathy, which can be largely reversed by the administration of recombinant CCSP. It remains unclear whether CCSP inhibits immunoglobulin-mediated lung inflammation.

Taken together, these data support the idea that CCSP has pleiotropic regulatory effects on innate and adaptive immune responses that promote pulmonary inflammation in the context of acute and chronic lung injury (Figure 1).

## ROLE OF CCSP IN HUMAN LUNG DISEASES

### Determinants of Circulating CCSP in Humans

CCSP has been extensively investigated in relation to human disease. Studies have measured CCSP mainly in serum and plasma, but also in other biospecimens such as sputum, bronchoalveolar lavage (BAL), nasal lavage, tracheal aspirate, urine, and others. CCSP/*SCGB1A1* gene expression in airway epithelial cells and CCSP/*SCGB1A1* genetic variation have also been assessed (see the section below titled CCSP Genotype in Disease).

Characterizing the role of CCSP in human disease requires first understanding the host and environmental factors that affect its gene expression and protein levels. Multiple genetic loci control a significant proportion of variability of circulating CCSP levels in humans (29–31). Circulating levels of CCSP increase from birth into adult life (32), possibly paralleling lung growth. In adulthood, plasma levels of CCSP are also influenced by changes in glomerular filtration rate, as CCSP is renally excreted (33). Girls have higher levels of circulating CCSP than boys in childhood (32), but in adult life the association of sex with CCSP level is less clear, possibly because of confounding effects by renal function and/or environmental exposures. Among the latter, the association of cigarette smoking with reduced circulating CCSP has been consistently reported (33, 34). CCSP levels in blood are reduced in response to many other chronic environmental exposures, including biomass smoke, air pollution, occupational exposures, and chlorination products (32, 35, 36). In general, acute exposures to factors that lead to epithelial injury result in a transient increase in circulating CCSP due to increased lung permeability, while chronic exposures lead to CCSP deficits, possibly due to club cell depletion and/or CCSP gene downregulation.

Notably, other factors augment CCSP and could be evaluated as potential therapeutic options in diseases characterized by CCSP deficits. A recent study (6) reported direct effects of retinoids on CCSP augmentation in vitro and significant effects of oral administration of vitamin A on circulating CCSP levels in vivo. Animal models have also suggested that the phosphodiesterase-4 inhibitor roflumilast can reverse CCSP downregulation in the airways in response to cigarette smoke (37) and that polydatin can increase CCSP expression in the lungs after lipopolysaccharide challenge (38). In addition, in vitro studies have indicated that glucocorticoids increase CCSP expression in lung epithelial cells (5).

Given the important role of CCSP in lung homeostasis, it is not surprising that CCSP is dysregulated in numerous lung diseases (Table 1) and likely plays an important role in pulmonary pathobiology. The following sections highlight examples of this dysregulation. We focus first on obstructive lung diseases, characterized mainly by airway inflammation and remodeling, and subsequently on restrictive lung diseases, which are primarily disorders of parenchymal (alveolar or interstitial) inflammation and/or fibrosis.

### CCSP in Obstructive Lung Diseases

Circulating CCSP is a strong biomarker of lung function. Population-based epidemiological studies (39–41) have shown that CCSP deficits are associated with indices of airflow limitation, including lower levels of forced expiratory volume in one second (FEV_1_), forced expiratory flow between 25% and 75% of vital capacity (FEF_25–75_), and the ratio of FEV_1_ to forced vital capacity (FEV_1_/FVC). Indeed, CCSP deficits have been observed among patients with COPD, which is characterized by irreversible airflow limitation. Serum CCSP levels were found to be lower in smokers with COPD than in smokers with no airflow limitation (42). Interestingly, patients with COPD also have lower CCSP expression in the airways (43) and CCSP deficits in sputum (44). In addition, several studies have reported an inverse association between CCSP and COPD severity: Airway CCSP immunostaining decreases with increasing COPD severity (43, 45), suggesting that club cell paucity may, at least in part, account for the reduction in CCSP production.

COPD can develop through at least two main lung function trajectories (46): one characterized by an accelerated FEV_1_ decline in adulthood and one by a low maximal lung function attained by young adult life. CCSP seems to play a role in both trajectories. Multiple studies have shown that low CCSP is associated with an accelerated FEV_1_ decline both among patients with COPD (47) and in the general adult population (40, 48). However, recent reports have also identified CCSP deficits in childhood as a risk factor for impaired lung function by adolescence (40) and into mid-adult life (41).

In clinical studies, circulating levels of CCSP were found to be lower in children (49) and adults (50) with asthma than in controls. Patients with asthma were also found to have low CCSP in BAL (51) and fewer CCSP-expressing cells in the airways (52). However, some population-based epidemiological studies did not find an association between asthma and circulating CCSP (39, 40), suggesting that CCSP deficits may characterize mainly persistent and/or more severe forms of asthma.

Only a few studies have investigated CCSP in cystic fibrosis, reporting significant airway CCSP deficits (53). Correlations between low BAL/sputum CCSP levels and airway inflammation (54) and disease severity (55) have also been identified. A report of children with cystic fibrosis (31) showed that lung function deficits are associated with remarkably low levels of circulating CCSP.

### CCSP in Restrictive Lung Diseases

In contrast to obstructive lung diseases, CCSP levels have been found to be increased in multiple restrictive lung diseases. CCSP concentrations were elevated in the BAL (56), as well as lung tissue (57, 58) and serum (56, 59), of patients with idiopathic pulmonary fibrosis compared to healthy controls. Patients with combined pulmonary fibrosis and emphysema had higher serum levels of CCSP than patients with emphysema alone or healthy controls (60). The higher levels of CCSP in the serum could simply reflect increased translocation of the protein in the context of lung injury. However, the protein amount in the fibrosed lung tissue was increased as well, implicating CCSP or club cells in the pathogenesis of fibrosis. Several mechanisms have been proposed: Some papers suggest a process of alveolar bronchiolization with proliferation and migration of club cells (61). A more recent paper assessing lung epithelial cells by single-cell RNA sequencing found an enriched population of CCSP^+^MUC5B^+^ cells in idiopathic pulmonary fibrosis, proposing that a change in club cell phenotype and number takes place (62).

Serum levels of CCSP were assessed in other fibrosing restrictive lung diseases and were found to be elevated in chronic hypersensitivity pneumonitis and connective tissue–related interstitial lung diseases (56), as well as systemic sclerosis (63, 64). Even though an early paper showed similar levels of serum CCSP in sarcoidosis patients compared to healthy controls (59), more recent studies demonstrated elevated levels correlating with lung disease severity (65, 66). BAL CCSP concentrations and numbers of CCSP^+^ cells detected by immunohistochemistry were not different (66), suggesting that sarcoidosis is not characterized by increased pulmonary CCSP production but rather increased leakage into the circulation. CCSP in interstitial lung diseases related to specific toxic exposures is less well understood. CCSP was elevated in the BAL of patients with silicosis (67) but serum levels were reduced (68). A small study of lung fibrosis after bleomycin exposure showed lower BAL CCSP levels and unchanged serum levels compared to controls (69).

Acute lung injury and ARDS, as well as respiratory distress syndrome in preterm infants, correlate with increased serum CCSP levels (70–72). Alveolar injury and/or mechanical ventilation may cause increased CCSP secretion. However, pulmonary levels of CCSP were found to be lower in ARDS than in hydrostatic or cardiogenic pulmonary edema (22). In either case, there is likely increased leakage of CCSP into the circulation due to disruption of the bronchoalveolar–blood barrier.

### CCSP in Post-Transplant Lung Diseases

Club cells and CCSP have been repeatedly implicated in the pathogenesis of chronic rejection after lung transplantation, referred to as chronic lung allograft dysfunction (CLAD). CLAD is identified by an irreversible decline in lung function and represents the most common cause of late death after lung transplantation (73). The most common CLAD manifestation is bronchiolitis obliterans syndrome (BOS), characterized by airway obstruction and histological findings of bronchiolitis obliterans (BO) with sparing of alveolar tissue. A less frequent but more severe manifestation of CLAD is restrictive allograft syndrome (RAS), distinguished by restrictive physiology and predominant parenchymal lung fibrosis, though BO is usually also observed (73).

Being primarily manifest as an obstructive lung disease, it is not surprising that CLAD is associated with reduced CCSP levels in BAL (74–77) as well as in serum (74). BAL CCSP was found to be inversely proportional to the frequency of neutrophils in BAL, suggesting a relationship between inflammation and CCSP depletion (74). Several studies also showed that lower levels of BAL CCSP precede CLAD diagnosis (74, 76), although this is not replicated in all papers (77), potentially due to significant interindividual variability in the protein levels and episodic acute changes in CCSP.

Decreased numbers of CCSP-expressing epithelial cells have been shown in airways affected by BO, suggesting a reduction in the actual frequency of club cells (75). In support of this concept, club cell ablation was found to cause BO in a mouse lung transplantation model (78). Club cells and CCSP thus appear to be protective against CLAD and BO. The process leading to BO after bone marrow transplantation, a manifestation of pulmonary graft-versus-host disease, is thought to be similar to CLAD. One study has assessed serum CCSP levels after bone marrow transplantation and did not find a correlation to BO (79). Nevertheless, a mouse model of BO post bone marrow transplantation has shown a protective role of CCSP (80).

Given the differences in CCSP levels between obstructive and restrictive lung diseases observed in the nontransplant context, there has been interest in comparing the CLAD phenotype RAS to BOS. Interestingly, one preliminary study, published in abstract form, found no significant difference in BAL CCSP levels between BOS and RAS, suggesting they are equally reduced in both phenotypes (81). However, larger studies are needed to confirm this, and further investigation of the role of club cells in the airway versus parenchymal fibrosing processes in CLAD is needed.

### CCSP GENOTYPE IN DISEASE

Human CCSP is encoded by a 3-kb single-copy gene, *SCGB1A1*, located on the long arm of chromosome 11, containing three exons and two introns. The most investigated CCSP variant in human disease is the G38A polymorphism (rs3741240) hallmarked by a guanine-adenine substitution at position 38 downstream from the transcription initiation site within the noncoding region of exon 1. Importantly, circulating or BAL CCSP levels have been shown to be significantly lower in subjects homozygous for the A allele as compared to heterozygotes or those homozygous for the G allele (49, 82, 83). Most studies evaluating the influence of CCSP variation on human disease risk have focused on its role in susceptibility to asthma or other atopic conditions (49, 83–88). Two meta-analyses reporting on the association between the G38A CCSP polymorphism and asthma susceptibility were published in 2013, demonstrating pooled odds ratios of asthma risk in A allele carriers of 1.29 and 1.62, respectively (89, 90). Extending these observations on CCSP genotype and asthma susceptibility, others have reported that the G38A polymorphism contributes to both asthma severity and attenuated response to glucocorticoid treatment (83, 87, 88). Notably, CCSP levels increased after glucocorticoid treatment in asthmatic patients with GG genotype, but not in those carrying an A allele. Consistent with these clinical observations, transfection assays designed to elucidate the transcriptional activity of the G38A genotype in a club-like cell line also demonstrated reduced CCSP gene expression (83).

While compelling, the observations from candidate gene investigations of CCSP genotype and asthma or atopy risk were not reproduced in every cohort and have not been recapitulated in larger genome-wide association studies (GWAS) of asthma, which presumably broadly captured common noncoding genetic variants such as the CCSP G38A polymorphism (91–93). This could be due to adjustments for multiple testing in such analyses, which may miss potentially informative gene loci unless extremely large sample sizes are studied, or due to the effect of CCSP genetic variation being mediated through gene–environment interactions. In fact, evidence does support the idea of a CCSP genotype–environment interaction. Specifically, while lung epithelial cells transfected with the CCSP G38A or 38G construct had similar baseline CCSP transcription levels, exposure to cigarette smoke extract more profoundly decreased CCSP transcription in 38A than in 38G transfected cells (94).

Similar to asthma studies, GWAS examining genetic determinants of COPD susceptibility have not implicated CCSP (95). However, many of the susceptibility loci identified in these studies have not resolved to functional variants. Thus, it remains uncertain if further work to resolve the causal variants underlying these COPD GWAS regions will elucidate findings relevant to CCSP expression. A sole candidate gene study in a small number of patients suggested that the CCSP G38A polymorphism was not associated with COPD risk (96). However, as mentioned above, CCSP levels are found to be reduced in blood, sputum, and BAL in association with COPD (29, 42–44, 97). Milne et al. (29) performed a GWAS and Mendelian randomization study of two large COPD cohorts using circulating CCSP level as the quantitative phenotype to investigate whether a causal relationship exists between circulating CCSP level and risk of COPD onset or progression. This analysis identified seven single-nucleotide polymorphisms (SNPs) that were independently associated with circulating CCSP levels in COPD patients. One of these was indeed the G38A polymorphism in CCSP (rs3741240) and explained the largest proportion of the variance in both cohorts. However, the other six identified SNPs were novel, including one additional intronic SNP (rs11231085) in CCSP that was the second largest contributor to explaining variance in circulating CCSP levels in these COPD subjects. Using the Mendelian randomization framework, the authors demonstrated a potential protective effect of genetically determined increased circulating CCSP levels on the risk of COPD or COPD progression (29). These findings suggest a possible causal role for CCSP deficit in COPD pathobiology.

Data on the relationship between CCSP variation and other lung diseases are less clear. The influence of the G38A polymorphism on sarcoidosis risk was examined in two studies generating conflicting results, perhaps attributable to differences in the ethnic composition of the cohorts under study (82, 98). This SNP was also found to be associated with lung function in smokers (48) and patients with cystic fibrosis (31). Additionally, two studies have assessed the role of donor or recipient G38A polymorphism on the risk for primary graft dysfunction (a form of acute lung injury) or CLAD after lung transplantation (99, 100): These studies showed that donor, but not recipient, genotype correlates with BAL CCSP levels early after transplantation and with the risk of post-transplant lung disease (99, 100).

Whether other clinically relevant CCSP variants exist remains unknown. In this regard, a recent study identified a novel *cis-*regulatory SNP, rs2509956, located downstream of the CCSP gene and in strong linkage disequilibrium with rs3741240 (101). Reporter assays confirmed that both rs2509956 and rs3741240 influenced CCSP gene expression in lung epithelial cells, and further studies suggested that rs2509956 could interact with a promoter region in CCSP, acting as an enhancer (101). Considering the strong linkage disequilibrium between this newly identified SNP and the widely studied G38A polymorphism, it is plausible that some of the effects of the G38A polymorphism identified in prior studies could, in part, be attributable to the influence of rs2509956; however, further studies are needed to explore this speculation.

## EVIDENCE FOR EFFICACY OF CCSP REPLACEMENT THERAPY

Replacement therapy with CCSP has proven effective in animal models of various lung diseases, including BO (80), ARDS (102), and lung inflammation (12). Additionally, no significant toxic side effects have been identified in large animal models, even at high doses, during intratracheal or intravenous administrations (102, 103).

Several studies have directly examined CCSP replacement in humans. A first pilot randomized placebo-controlled trial used rhCCSP replacement in the treatment of premature infants with respiratory distress syndrome (104). A total of 22 patients were enrolled in the study, receiving placebo or rhCCSP (1.5 mg/kg or 5 mg/kg) intratracheally. The rhCCSP was generally well tolerated, and it appeared to reduce some indices of lung inflammation (e.g., total cell counts, neutrophils, total protein, interleukin-6) in tracheal aspirate, but it did not significantly improve clinical outcomes such as oxygen usage or ventilator days (which were prolonged in the 5 mg/kg treatment arm). Of the 22 infants enrolled, there were two deaths in the rhCCSP treatment groups and none in the placebo group. Follow-up at 6 months, corrected for gestational age, indicated that treatment with rhCCSP was associated with a reduced number of respiratory-associated hospitalizations and decreased treatment with bronchodilators (105).

Based on potential longer-term benefits of rhCCSP in the treatment of bronchopulmonary dysplasia, a follow-up study was conducted that included 88 neonates, again comparing rhCCSP at doses of 1.5 mg/kg or 5 mg/kg to placebo (105). The study used a multicenter double-blind placebo-controlled randomized dose-escalation design. The primary outcome was survival to 12 months without any chronic pulmonary insufficiency of prematurity (CPIP) as assessed by parent-reported outcomes related to respiratory visits or symptoms. There was no difference in the development of the primary outcome in the placebo group as compared to either treatment group, with very few patients alive and free from CPIP in either group. Rates of adverse events, severe adverse events, and death were comparable among the treatment arms. There were no significant differences between groups for individual CPIP components or combinations thereof.

A different approach and patient population were employed in a study that included adult male patients with allergic rhinitis treated with intranasal delivery of rhCCSP (0.56 mg per nasal cavity) once daily for 7 days in a double-blind placebo-controlled crossover design (106). Between placebo- and CCSP-treated subjects, no differences were observed in clinically significant measures of allergic outcomes or in biomarkers of eosinophilic or neutrophil activity.

In summary, the previous clinical experience with CCSP replacement did not demonstrate clear clinical benefit. However, the studies are limited to treatment in two very specific populations and to delivery using intratracheal or nasal instillation of rhCCSP; they included relatively small sample sizes and used single-dose or limited-duration treatment. Intriguing preclinical and clinical results in COPD, asthma, and CLAD suggest that reduced CCSP is a biomarker of disease or disease progression, and mechanistic studies have demonstrated the direct or indirect anti-inflammatory effects of CCSP on immune and epithelial cells in the lung. In light of these findings, further interventional studies using CCSP replacement in these conditions should be pursued. However, the optimal delivery route, dosing, and dose frequency will have to be defined. Additionally, indirect approaches for endogenous CCSP augmentation may be of benefit and merit further investigation (see the section titled Determinants of Circulating CCSP in Humans).

## CONCLUSION

Multiple studies have assessed CCSP levels in lung samples or peripheral blood, showing that CCSP is generally decreased in obstructive and increased in restrictive lung diseases. While environmental and injurious factors can have opposing effects on CCSP, rendering the levels difficult to interpret in certain contexts, CCSP is overall an important biomarker of airway epithelial health and lung functional status. The exact diagnostic value for clinical application will need rigorous validation, as each specific disorder will likely require its own reference values for estimation of disease severity and risk stratification.

While correlation with lung function and disease severity is intriguing, this information does not necessarily imply causality, and more mechanistic studies are needed to fully understand the role of CCSP in specific pulmonary diseases. Genetic studies of CCSP allude to causal inference, further supporting a protective role of CCSP. Additionally, while CCSP levels are often equated with the health and numbers of club cells, potential extrapulmonary and extra–club cell sources of CCSP need further clarification. Finally, the numerous studies that show beneficial effects of CCSP in animal models of diseases merit further follow-up and exploration of its therapeutic potential in humans. Future human interventional studies focusing on CCSP should include relevant mechanistic endpoints to allow a better understanding of detailed biological effects in vivo.

## Figures and Tables

**Table 1:** CCSP genetic polymorphism associations and CCSP levels in the lung tissue, bronchoalveolar lavage, serum, or plasma in obstructive and restrictive lung diseases

	Evidence to support CCSP genotype association	Lung tissue/BAL CCSP level^a^	Serum/plasma CCSP level^a^	Some illustrative references
***Obstructive lung diseases***				
Lung function levels and/or decline	Unknown	Unknown	↓	39–41, 47, 48, 97
COPD status, severity or progression	rs3741240 rs11231085	↓	↓	29, 42–45
Asthma	rs3741240	↓	↔↓	39, 40, 49–52, 87
Cystic fibrosis status, severity, or progression	rs3741240	↓	↓	31, 53–55
Chronic lung allograft dysfunction	Donor rs3741240	↓	↓	74, 75, 100
Pulmonary graft-versus-host disease	Donor rs3741240	Unknown	↔	79, 99
***Restrictive lung diseases***				
Idiopathic pulmonary fibrosis	Unknown	↑	↑	56–59
Combined pulmonary fibrosis and emphysema	Unknown	Unknown	↑	60
Chronic hypersensitivity pneumonitis	Unknown	Unknown	↑	56
CTD-ILD	Unknown	Unknown	↑	56
Systemic sclerosis with pulmonary fibrosis	Unknown	Unknown	↑	63, 64
Sarcoidosis	rs3741240 (equivocal)	↔	↔↑	65, 66, 82, 98
Bleomycin	Unknown	↓	↔	69
Silicosis	Unknown	↑	↓	67, 68
Infant respiratory distress syndrome	Unknown	Unknown	↑	72
ARDS	Unknown	↓↔	↑	70
COVID-19 ARDS	Unknown	Unknown	↑	71

a Cells that may contribute to lung/BAL CCSP content are airway club cells, alveolar epithelial cells, and macrophages. Though lung cells are thought to be the primary contributors to circulating CCSP content, CCSP has also been identified in human uterine, thymus, prostate, and pituitary gland tissues and may contribute to circulating levels.

Abbreviations: ARDS, acute respiratory distress syndrome; BAL, bronchoalveolar lavage; CCSP, club cell secretory protein; COPD, chronic obstructive pulmonary disease; COVID-19, coronavirus disease 2019; CTD-ILD, connective tissue disease-related interstitial lung disease.
